# The Pasteur Institute

**Published:** 1890-03

**Authors:** A. Dagenais

**Affiliations:** Buffalo, N. Y.


### THE PASTEUR INSTITUTE.

Translated from *L'Abeille Médicale.*

By A. DAGENAIS, M. D., Buffalo, N. Y.

FIVE months have elapsed since any death has taken place at the institute, the last being Jose Manuel, treated with inoculation, in July, who died on August 21, 1889. Since that period, 850 new cases have been treated; from this number, none have died. As any one can see, this statistic of the hydrophobia, so encouraging in its debut, has been perfected by practice, and the actual results exceed the hopes that were entertained at its origin. There are two main factors for these happy results—first, in general, the patients come under the treatment sooner than before; second, some modifications have taken place in the location of the inoculations—for instance, the quality of the liquid injected is much larger than before; besides, when the wounds are of a very dangerous character, like bites, repeated and deep, or affecting the face, on the cranium, then the marrows of a virulent strength, as the one of three days' standing, are injected, not only during one day, but during two consecutive days.

